# Physical Training Regulates Mitochondrial Parameters and Neuroinflammatory Mechanisms in an Experimental Model of Parkinson's Disease

**DOI:** 10.1155/2015/261809

**Published:** 2015-09-10

**Authors:** Talita Tuon, Priscila S. Souza, Marcela F. Santos, Fernanda T. Pereira, Giulia S. Pedroso, Thais F. Luciano, Claudio T. De Souza, Rafael C. Dutra, Paulo C. L. Silveira, Ricardo A. Pinho

**Affiliations:** ^1^Laboratory of Exercise Biochemistry and Physiology, Graduate Program in Health Sciences, Health Sciences Unit, Universidade do Extremo Sul Catarinense, 88806-000 Criciúma, SC, Brazil; ^2^Laboratory of Autoimmunity and Immunopharmacology, Campus Araranguá, Universidade Federal de Santa Catarina, 88900-000 Araranguá, SC, Brazil

## Abstract

This study aimed to evaluate the effects of two different protocols for physical exercise (strength and aerobic training) on mitochondrial and inflammatory parameters in the 6-OHDA experimental model of Parkinson's disease. Six experimental groups were used (*n* = 12 per group): untrained + vehicle (Sham), strength training + vehicle (STR), treadmill training + vehicle (TTR), untrained + 6-OHDA (U + 6-OHDA), strength training + 6-OHDA (STR + 6-OHDA), and treadmill training + 6-OHDA (TTR + 6-OHDA). The mice were subjected to strength or treadmill training for 8 weeks. PD was induced *via* striatal injection of 6-OHDA 24 h after the last exercise session. Mice were euthanized by cervical dislocation and the striatum and hippocampus were homogenized to determine levels of tyrosine hydroxylase (TH), nuclear factor kappa B (NF-*κ*B) p65, and sirtuin 1 (Sirt1) by western blot; tumor necrosis factor-*α* (TNF-*α*), interleukin-1*β* (IL-1*β*), IL-17, interferon-*γ* (IFN-*γ*), and transforming growth factor *β*1 (TGF-*β*1) levels by ELISA; NO content; and complex I (CI) activity. STR + 6-OHDA mice had higher TH levels and CI activity and lower NF-*κ*B p65 and IFN-*γ* levels in the striatum compared to U + 6-OHDA mice, while TTR + 6-OHDA mice had higher Sirt1 levels and CI activity in both the striatum and the hippocampus, compared to U + 6-OHDA mice. Strength training increased CI activity and TH and Sirt1 levels and reduced NO, NF-*κ*B p65, TNF-*α*, IFN-*γ*, IL-1*β*, and TGF-*β*1 levels in 6-OHDA mice, while treadmill exercise increased CI activity and NO, TH, and Sirt1 levels and reduced NF-*κ*B p65, TNF-*α*, IFN-*γ*, and IL-1*β* levels. Our results demonstrated that both treadmill training and strength training promote neuroprotection, possibly by stimulating Sirt1 activity, which may in turn regulate both mitochondrial function and neuroinflammation *via* deacetylation of NF-*κ*B p65. Changes in nitric oxide levels may also be a mechanism by which 6-OHDA-induced inflammation is controlled.

## 1. Introduction

Parkinson's disease (PD) is a progressive neurodegenerative disease with a global prevalence of 1%–3% in the population aged over 55 years [1, 2]. Although the etiology of PD is poorly understood, many factors are associated with susceptibility and disease manifestation. The symptoms of PD result from the death of dopamine-producing cells in the substantia nigra, which has been linked to several mechanisms including inflammation and mitochondrial dysfunction [3–5].

Several recent experimental studies have indicated a role for mitochondrial dysfunction [6–9] and inflammation [10–14] in PD. The concept that mitochondrial abnormalities are related to the pathogenesis of PD emerged from evidence for decreased mitochondrial complex I activity and increased activity of the *α*-ketoglutarate dehydrogenase complex in patients with PD [7, 15, 16]. In addition, inflammation plays an important role in the neurodegeneration of nigrostriatal neurons [17, 18]. Studies have demonstrated high levels of proinflammatory cytokines in the nigrostriatal regions in patients with PD [12–14, 19]. The presence of these proinflammatory mediators contributes to increased microglial activation and appears to amplify and maintain neuroinflammation. In addition, microglial activation can be toxic and induce enzymes such as myeloperoxidase, cyclooxygenase-2, nitric oxide synthase, and NADPH oxidase, thereby increasing the oxidation of biomolecules [20]. Therefore, reducing the activity of these enzymes reduces neuronal loss and the signs and symptoms of the disease [21–23].

Treatment with levodopa and dopamine agonists effectively manages the early motor symptoms of PD; however, the progressive and irreversible nature of the disease, where dopaminergic neurons continue to die, makes these drugs ineffective for treating the symptoms of involuntary movement. Some forms of rehabilitation, such as physical exercise, have been effective at alleviating these symptoms through neuroprotection. Previous studies by our group have shown that exercise modulates the neurochemical status of the striatum and hippocampus in a rodent model of PD, most likely by increasing the levels of regulatory neurotrophins and reducing oxidative stress [24, 25]; however, these results may be related to the characteristics of the exercise being performed. The effects of exercise on neuroinflammation and mitochondrial dysfunction in PD are not clearly understood; therefore, this study sought to investigate the effects of two types of physical training on protein levels involved in mitochondrial biogenesis and inflammatory function in the striatum and hippocampus of 6-OHDA-lesioned mice.

## 2. Material and Methods

### 2.1. Animals

Adult male C57BL/6 mice (age: 2 months; weight: 25–30 g) were obtained from our own breeding colony. Mice were housed five per cage, on a 12/12 h light/dark cycle (lights on at 07:00) with free access to food (Nuvilab CR1, NuvitalNutrientes S/A, Brazil) and water. All experimental procedures were carried out in accordance with the Brazilian Guidelines for the Care and Use of Animals for scientific and didactic purposes (DOU 27/5/13, MCTI, p.7) and the study was approved by the local ethics committee. Six experimental groups were used (*n* = 12 in each group): untrained + vehicle (Sham), strength training + vehicle (STR), treadmill training + vehicle (TTR), untrained + 6-OHDA (U + 6-OHDA), strength training + 6-OHDA (STR + 6-OHDA), and treadmill training + 6-OHDA (TTR + 6-OHDA).

### 2.2. Exercise Protocols

#### 2.2.1. Treadmill Training

All mice were habituated on a nine-channel, motor-driven treadmill at a speed of 10 m·min^−1^ for 10 min/day for 1 week to reduce their stress in response to the new environment. The mice did not receive any stimulus to run. The exercise groups performed an incremental running program to obtain progressive levels of intensity (13–17 m·min^−1^, no incline) on 3 or 4 days/week for 8 weeks, for a total period of 60 days. Each session was 50 min in duration and there was a 48 h interval between sessions.

#### 2.2.2. Strength Training

This exercise entailed the mice climbing a 1 m ladder with a 2 cm grid inclined at 85° [26]. Mice were familiarized with the exercise for 3 days. Three days later, resistance training was begun using cylinders containing weights that were attached to the base of the tail of the mouse with foam tape. Briefly, the cylinders were fastened to the tail by wrapping the upper portion of the tail (2-3 cm from the proximal end) with Velcro on top of the foam tape. Then, the initial weights (50% of body weight) were inserted into the cylinders. The mouse was then positioned at the base of the climbing apparatus and motivated to climb the ladder using a grooming action to the tail. The weight attached to the tail was increased gradually from 50% to 100% throughout the 8 weeks of training: 1st and 2nd week, 50%; 3rd and 4th week, 60%; 5th and 6th week, 80%; 7th and 8th week, 100%. Three sets of five repetitions, with a 1 min rest between repetitions and 2 min rest between sets, were performed for 3 or 4 days/week. Each session was 40–50 min in duration, with a 48 h interval between sessions. When the mice reached the top of the ladder, they were allowed to recover in a resting area. This procedure was repeated until the mice finished three sets of training or they failed to climb the entire length of the ladder. The mice were manually stimulated to provide motivation to climb when necessary.

#### 2.2.3. Surgical Procedures

Twenty-four hours after the final physical training session, mice were anesthetized with Equithesin (3 mL/kg, i.p.) and placed on a stereotaxic frame. Eight micrograms of 6-OHDA was administered to each mouse (2 *μ*g/*μ*L prepared in 0.2% ascorbic acid and 0.9% saline)* via* unilateral injections into the terminal region of the striatum. The coordinates were AP = +1 mm; ML = ±1.7 mm; DV = −2.9 mm from bregma [27]. The 6-OHDA was injected* via* a Hamilton syringe attached to an infusion pump (BI Insight 2000) at a rate of 0.5 *μ*L/min for 8 min. Following the injection, the needle was left in place for 3 min before slowly retracting to prevent reflux. Control (Sham) animals received the same volume of vehicle solution (0.2% ascorbic acid and 0.9% saline) using an identical procedure.

#### 2.2.4. Euthanasia and Tissue Collection

Mice were killed by cervical dislocation, and the striatum and hippocampus were surgically removed. One aliquot of each sample was homogenized in a buffer containing 1% Triton X-100, Tris 100 mM (pH 7.4), sodium pyrophosphate 100 mM, EDTA 100 mM, sodium vanadate 10 mM, phenylmethanesulfonyl fluoride 2 mM, and aprotinin 0.1 mg/mL at 4°C for intracellular protein analyses by western blot. The homogenate was centrifuged at 11000 rpm for 40 min to remove insoluble material. The supernatant was collected and the protein concentration was determined using the Bradford method [28]. Another aliquot was processed and stored at −70°C for later analysis by ELISA.

### 2.3. Bioassay Measurements

#### 2.3.1. Complex I Activity and NO Production

The activity of complex I was determined as previously described [29]. This method is based on NADH dehydrogenase activity of the NADH-dependent reduction of ferricyanide at 420 nm. The activity of complex I was measured before the addition of rotenone (20 g/mL) and the absorbance was monitored for a further 5 min. The activity of complex I was determined as the sensitivity to rotenone and was expressed as nmol/min/mg protein. NO production was estimated spectrophotometrically, based on nitrite generation. Samples were incubated with Griess reagent (1% sulfanilamide in 0.1 M HCl and 0.1%* N*-(1-naphthyl)ethylenediamine dihydrochloride) at room temperature for 10 min and the absorbance was measured at 540 nm using a microplate reader [30].

#### 2.3.2. Protein Analysis by Immunoblotting

Proteins were denatured by boiling in sample buffer containing 100 mM DTT [31]. After this, 0.2 mg of protein from each sample was separated by SDS-PAGE and transferred onto nitrocellulose membranes. The membranes were incubated with the following antibodies: anti-tyrosine hydroxylase (TH, SC25269), anti-sirtuin 1 (SIRT1, SC15404), and anti-nuclear factor kappa-light-chain-enhancer of activated B cells p65 (NF-*κ*B p65 and SC71675). Antibodies were supplied by Santa Cruz Biotechnology (Santa Cruz, CA, USA). Chemiluminescent detection was performed with horseradish peroxidase-conjugate secondary antibodies (Thermo Scientific, Rockford, IL, USA). The light signal was captured on X-ray film exposure for visualization of protein bands. The product of the area and intensity (area X intensity) of the bands were used for comparison intensities between the apparent bands in the autoradiographs. The bands were quantified by densitometry and expressed as arbitrary units using Scion Image software (Scion Image Software, ScionCorp, Frederick, MD, USA). These values were then directly compared. The original membrane was stripped and probed with anti-*β*-actin (Ab8227) as a loading control.

#### 2.3.3. Determination of Interleukin Concentrations

Striatal and hippocampal samples were homogenized in phosphate buffer containing 0.05% Tween 20, 0.1 mM phenylmethylsulfonyl fluoride, 0.1 mM benzethonium chloride, 10 mM ethylenediaminetetraacetic acid, and 20 IU aprotinin. The homogenate was centrifuged at 3000 ×g for 10 min and the supernatants were stored at −70°C for later analysis. The concentration of tumor necrosis factor alpha (TNF-*α*, DY410), interleukin-1 beta (IL-1*β*, DY401), interleukin-17 (IL-17, DY421), interferon gamma (IFN-*γ*, DY485), and transforming growth factor-beta 1 (TGF-*β*1, DY1679) were evaluated by enzyme-linked immunosorbent assay (ELISA), using kits from R & D Systems according to the manufacturer's recommendations.

### 2.4. Statistical Analysis

All data are presented as mean ± standard error of the mean (SEM). Differences between experimental groups were determined using two-way analysis of variance (ANOVA) followed by Tukey's post hoc test. A *P* value of <0.05 was considered to be statistically significant.

## 3. Results

### 3.1. Neurodegeneration Marker

**Figure 1:**
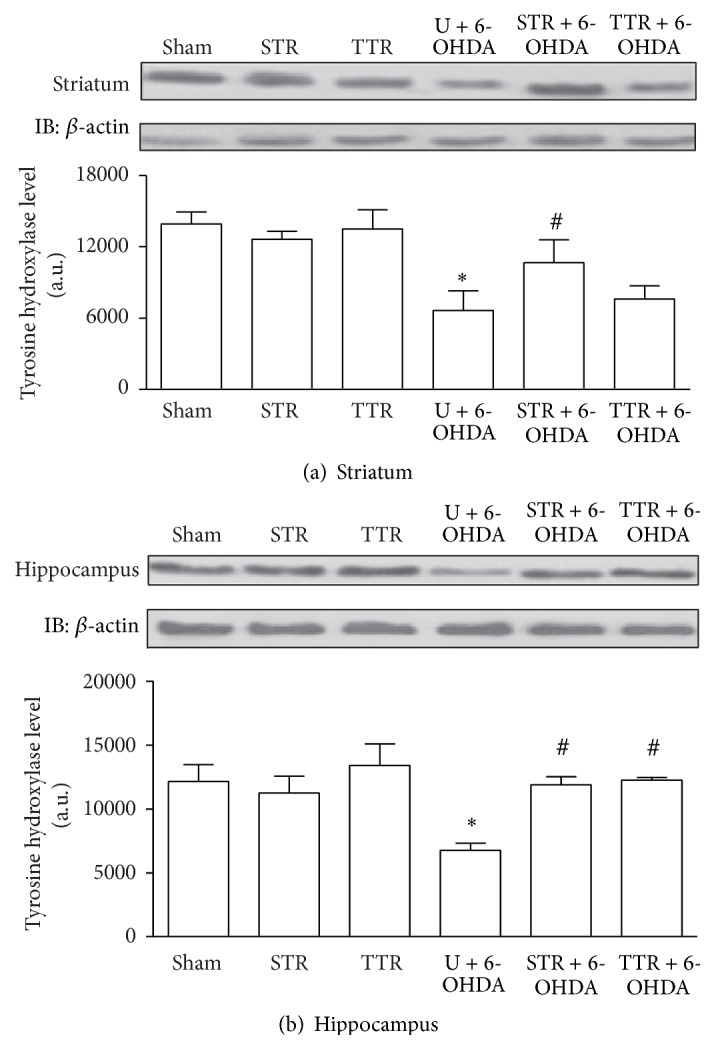
The effects of two physical training protocols on TH levels (a and b) in the striatum and hippocampus of mice exposed to 6-OHDA. Protein levels were assayed by western blotting. Values are expressed as mean ± SEM (*n* = 3). ^*∗*^ *P* < 0.05, Sham* versus* U + 6-OHDA; ^#^ *P* < 0.05, U + 6-OHDA* versus* training groups + 6-OHDA. Untrained + vehicle (Sham), strength training + vehicle (STR), treadmill training + vehicle (TTR), untrained + 6-OHDA (U + 6-OHDA), strength training + 6-OHDA (STR + 6-OHDA), and treadmill training + 6-OHDA (TTR + 6-OHDA).

TH levels in the striatum and hippocampus are presented in Figures 1(a) and 1(b), respectively. Both cerebral structures showed lower TH levels in the U + 6-OHDA group compared with the Sham group. However, strength training increased the levels of TH in the striatum; accordingly, TH levels were higher in the STR + 6-OHDA group than in the U + 6-OHDA group. Both training models showed similar results in the hippocampus.

**Figure 2:**
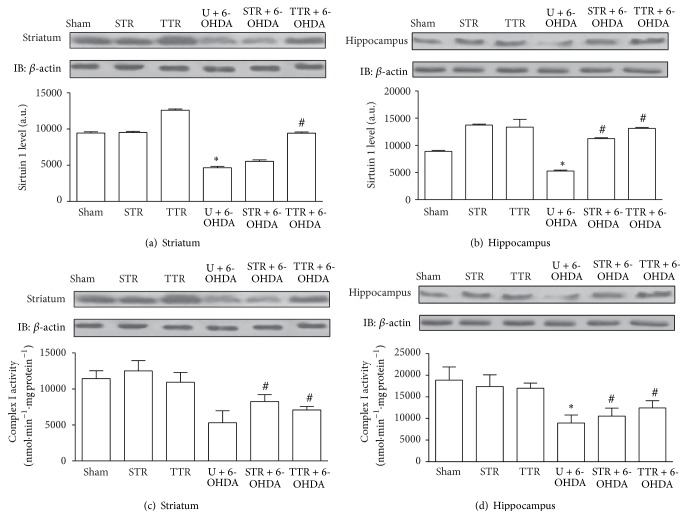
The effects of two physical training protocols on Sirt1 levels (a and b, *n* = 3) and complex I activity (c and d, *n* = 6) in the striatum and hippocampus of mice exposed to 6-OHDA. Hippocampus and striatum protein extracts containing 0.2 mg total protein were separated by SDS-PAGE, transferred to nitrocellulose membranes, and probed with anti-Sirt1 antibody. Complex I activity was assayed by measuring the NADH-dependent reduction of ferricyanide. Values for Sirt1 are presented as mean area × intensity of bands ± SEM (arbitrary units), and complex I activity is expressed as mean ± SEM. ^*∗*^ *P* < 0.05, Sham* versus* U + 6-OHDA; ^#^ *P* < 0.05, U + 6-OHDA* versus* training groups plus 6-OHDA. Untrained + vehicle (Sham), strength training + vehicle (STR), treadmill training + vehicle (TTR), untrained + 6-OHDA (U + 6-OHDA), strength training + 6-OHDA (STR + 6-OHDA), and treadmill training + 6-OHDA (TTR + 6-OHDA).

*Mitochondrial Parameters*. There were similar levels of Sirt1 in the striatum and hippocampus. As shown in Figures 2(a) and 2(b), a decrease in Sirt1 was observed in the U + 6-OHDA group compared with the Sham group. However, only treadmill training attenuated this decrease in the striatum. Both treadmill training and strength training attenuated the decrease in Sirt1 in the hippocampus. There was a significant decrease in complex I activity in the U + 6-OHDA group compared with the Sham group (Figures 2(c) and 2(d)). However, both striatal and hippocampal complex I levels increased significantly in the STR + 6-OHDA and TTR + 6-OHDA groups compared with the U + 6-OHDA group.

### 3.2. Molecules Involved in the Release and Synthesis of Cytokines (NO and NF-*κ*B Levels)

**Figure 3:**
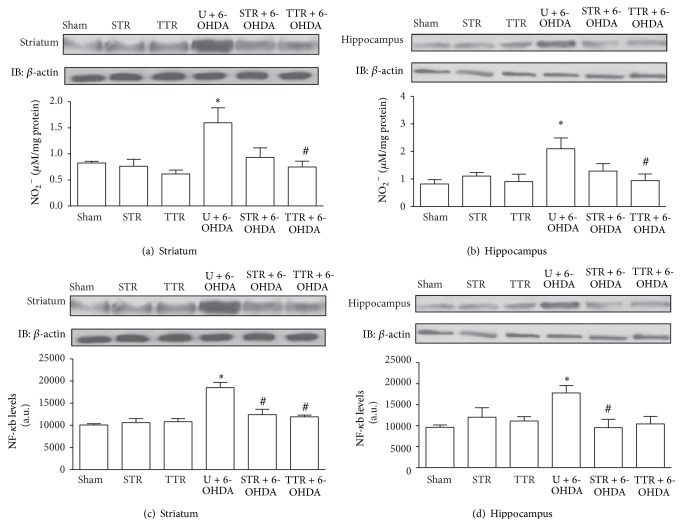
The effects of two physical training protocols on nitric oxide (a and b, *n* = 6) and NF-*κ*B p65 (c and d, *n* = 3) levels in the striatum and hippocampus of mice exposed to 6-OHDA. For western blotting, hippocampus and striatum protein extracts containing 0.2 mg total protein were separated by SDS-PAGE, transferred to nitrocellulose membranes, and probed with anti-NF-*κ*B-p65 antibody. Nitrite generation by NO was assayed spectrophotometrically. Values for NF-*κ*B p65 are presented as mean area × intensity of bands ± SEM (arbitrary units), and oxide nitric levels are expressed as mean ± SEM. ^*∗*^ *P* < 0.05, Sham* versus* U + 6-OHDA; ^#^ *P* < 0.05, U + 6-OHDA* versus* training groups plus 6-OHDA. Untrained + vehicle (Sham), strength training + vehicle (STR), treadmill training + vehicle (TTR), untrained + 6-OHDA (U + 6-OHDA), strength training + 6-OHDA (STR + 6-OHDA), and treadmill training + 6-OHDA (TTR + 6-OHDA).

Striatal and hippocampal NO were increased in the U + 6-OHDA group compared with the Sham group. When mice were subjected to treadmill physical training (TTR + 6-OHDA), NO levels were significantly decreased compared with the U + 6-OHDA group (Figures 3(a) and 3(b)) in the hippocampus and striatum. The levels of total NF-*κ*B p65 protein in the striatum and hippocampus increased in the U + 6-OHDA group. In the striatum, a significant decrease was observed in both the STR + 6-OHDA and TTR + 6-OHDA trained groups (Figure 3(c)), while, in the hippocampus, a significant decrease in the levels of total NF-*κ*B p65 protein was detected only in the STR + 6-OHDA group compared with the U + 6-OHDA group (Figure 3(d)).

### 3.3. Proinflammatory Cytokines

**Figure 4:**
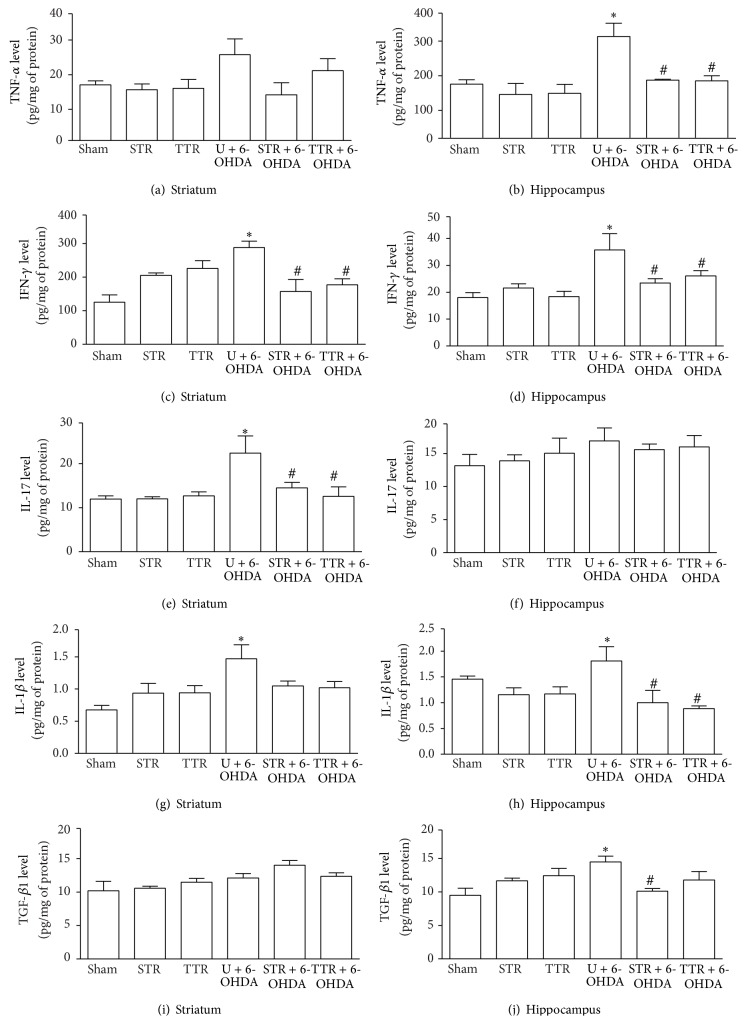
The effects of two physical training protocols on TNF-*α* (a and b), IFN-*γ* (c and d), IL-17 (e and f), IL-1*β* (g and h), and TGF-*β* (i and j) levels in the striatum and hippocampus of mice exposed to 6-OHDA. Protein levels of these cytokines were assayed using ELISA kits. Values are expressed as mean ± SEM (*n* = 6). ^*∗*^ *P* < 0.05, Sham* versus* U + 6-OHDA; ^#^ *P* < 0.05, U + 6-OHDA* versus* training groups plus 6-OHDA. Untrained + vehicle (Sham), strength training + vehicle (STR), treadmill training + vehicle (TTR), untrained + 6-OHDA (U + 6-OHDA), strength training + 6-OHDA (STR + 6-OHDA), and treadmill training + 6-OHDA (TTR + 6-OHDA).

There were high levels of proinflammatory cytokines in the mice that underwent either type of physical training. There was no significant difference in the level of TNF-*α* in the striatum of TTR and ST mice compared with the Sham group (Figure 4(a)). However, there was a significant increase in the level of TNF-*α* in the hippocampus of the U + 6-OHDA group compared with the Sham group. When mice were subjected to either type of physical training, a significant decrease in the level of TNF-*α* was observed (Figure 4(b)). IFN-*γ* levels in the striatum and hippocampus were increased in the groups exposed to 6-OHDA PD, but the animals that had been subjected to either training model showed a significant decrease in both the hippocampus and striatum (Figures 4(c) and 4(d)). IL-17 levels were increased in the striatum in the U + 6-OHDA group compared with the Sham group (Figure 4(e)) and a decrease in IL-17 levels was observed when the animals were subjected to either type of physical training (Figure 4(e)). No significant difference in the levels of IL-17 was observed in the hippocampus between groups (Figure 4(f)). IL-1*β* levels in the striatum and hippocampus were increased in the U + 6-OHDA group compared with the Sham group. When 6-OHDA-lesioned mice were subjected to physical training, a significant decrease in IL-1*β* levels was observed in the hippocampus (Figure 4(h)). There was no significant difference in the level of TGF-*β*1 in the striatum between groups (Figure 4(i)). There was an increase in TGF-*β*1 levels in the hippocampus of the U + 6-OHDA group. TGF-*β*1 levels were significantly reduced when 6-OHDA-lesioned mice were subjected to strength training (Figure 4(j)).

## 4. Discussion

This study was designed to investigate the effects of two different physical training protocols (aerobic treadmill training and strength training) on markers of mitochondrial function and regulatory mechanisms for neuroinflammation in the striatum and hippocampus of mice exposed to the 6-OHDA experimental model of PD. The striatum and hippocampus are two cerebral structures that respond positively to the neuroprotective effects of physical exercise, particularly in relation to mitochondrial function, oxidative stress, and neuroinflammation [8, 24, 32–34].

The efficacy of the experimental model was determined by measuring the TH levels, which is indicative of the neurodegeneration induced by 6-OHDA [35, 36]. TH deficiency was observed in the striatum and hippocampus of 6-OHDA-injected mice; however, this deficiency was effectively attenuated when the mice had previously undergone either treadmill or strength training. Similar results have been observed in previous studies using different training weights and similar intensities [37–39]. In addition, previous studies of our group [24, 25] have shown that physical training exerts effects on rotational test which produces well-defined and stable behavioral effects. We have demonstrated an increase in the number of turns in animals with Parkinson's disease, but the animals' exposure to exercise shows the number of turns as being near to the control group. This response to exercise reflects a protective effect on dopaminergic neurons in the striatum, particularly on dopamine production and this result is reinforced by the response of TH to exercise observed in the present study. Taken together, these results suggest that physical training, regardless of the type, contributes to the prevention and treatment of PD in experimental models. Several factors have been described as major mediators of exercise-induced neuroprotection in PD, but there is strong evidence for changes in neurotrophic factors [39, 40]. The underlying mechanisms induced by physical training on experimental models, such as a functional improvements in neurons that survive the 6-OHDA exposure and activation of endogenous neurogenesis, are responsible for the neuroprotection. These factors may be dependent on mitochondrial function and neuroinflammatory mechanisms [41].

Several experimental studies have shown that there is mitochondrial dysfunction in PD, particularly resulting from a reduction in mitochondrial complex I activity [42] and altered levels and activity of proteins involved in the cellular redox environment [43, 44]. Our results showed decreased complex I activity and Sirt1 levels in the hippocampus and striatum of 6-OHDA-lesioned mice. These levels were significantly restored in mice that had been previously subjected to either physical training model, except in the STR + 6-OHDA group at which Sirt1 levels were not significantly increased in the striatum. Complex I is a major component of the electron transport chain. It comprises a set of functional molecules with the ability to utilize the NAD^+^/NADH ratio to translocate protons across the inner membrane and promote oxidative ATP synthesis [45]. The involvement of this complex has been demonstrated in the 6-OHDA experimental model [42], making ATP-dependent cells more vulnerable to apoptosis and contributing to the production of reactive oxygen species. This leads to the dysfunction and death of cells during the development of PD [20].

Exercise may affect brain respiratory chain complexes, particularly complex I,* via* changes in proteins that regulate mitochondrial function. Sirt1, for example, regulates metabolic processes in different tissues and has been associated with neuroprotection [43, 46]. It is involved in regulatory processes such as the NAD^+^-dependent deacetylation of proteins, namely, heat shock factor protein 1 [47], NF-*κ*B [48], and PGC-1*α* [49], which alter the redox environment of the brain and reduce the aggregation of *α*-synuclein [47]. Therefore, changes in complex I can contribute to a change in the NAD^+^/NADH ratio and raise compensatory and regulatory Sirt1 levels from endogenous stimuli or by physical training, which is most likely dependent on the activation of PGC1-*α* [38, 44, 49].

Neuroinflammation is important in the pathogenesis of PD [22]; in particular NF-*κ*B has an important role in this process [48]. NF-*κ*B is located in the nuclei of neurons and glial cells. Interestingly, studies using animal models of PD suggest a neuroprotective role for NF-*κ*B due to its ability to regulate genes involved in the production of free radicals and mitochondrial function. However, what factors determine whether the activation of NF-*κ*B is beneficial or harmful to neurons in PD remain unclear.

The results presented here show that the levels of total NF-*κ*B p65 protein in hippocampal and striatal were elevated in animals injected with 6-OHDA, and these increases were significantly attenuated when the mice had previously undergone either treadmill or strength training. This suggests a possible neuroprotective mechanism regulated by the NF-*κ*B. One possible mechanism that could promote regulation by NF-*κ*B may be the increased levels of Sirt1 that were also observed in the present study. SIRT1, an NAD(+)-dependent protein deacetylase, has been shown to suppress NF-*κ*B signaling through deacetylation of the p65 subunit of NF-*κ*B leading to the suppression of proinflammatory interleukins [48].

The high levels of proinflammatory cytokines that were observed in this study, such as IFN-*γ*, TNF-*α*, IL-1*β*, IL-17, and TGF-*β*, have been associated with PD [50] and can induce neuronal damage through a variety of mechanisms, including the production of reactive oxygen species [51]. Surprisingly IL-17 was not changed in the hippocampus, and the reasons for this are not clearly explained by the literature. One possible explanation for the IL-17 results is that, within the IL-17 family, IL-17D is the interleukin that is more highly expressed in the nervous system. Thus, the results found in the hippocampus may be due to the use of nonspecific ELISA kit to evaluate the total levels of IL-17. Proinflammatory cytokines and factors released by dopaminergic neurons amplify and sustain neuroinflammation and immune responses, leading to the irreversible destruction of these neurons in the nervous system [52]. Under normal physiological conditions, these cytokines are expressed at low levels in the hippocampus and striatum but they can be induced to high levels by neurodegenerative stimuli [51].

Another possible mechanism responsible for the elevated levels of proinflammatory cytokines in the hippocampus and striatum of PD animal models could be increased nitric oxide production. Glial cells can generate nitric oxide at neurotoxic levels following stimulation from high levels of IFN-*γ* [53, 54], which induces CD23 expression following its release from microglia [21, 55]. CD23 can induce the production of nitric oxide synthase [56] and nitrates, which can mediate the synthesis of cytokines. Thus, the effects of exercise on inflammatory mediators could be directly associated with the regulation of nitric oxide synthesis and thereby contribute to a reduction in proinflammatory proteins. Previous studies have shown that treadmill training reduces levels of neuronal and inducible nitric oxide synthase in the striatum and hippocampus of experimental models of PD by reducing the activity of microglia [57, 58]. Taken together, the results of this study validate the positive effects of physical training on brain pathology in the 6-OHDA mouse model of PD.

## 5. Conclusion

In summary, 6-OHDA induced different changes in the hippocampus and striatum, particularly in the levels of IL-17 and TGF-*β*, which should be investigated further. However, both structures responded similarly to the different models of physical training used in this study. Both treadmill and strength training promoted neuroprotection and this may be* via* stimulation of Sirt1, which can regulate both mitochondrial function and neuroinflammation by the deacetylation of NF-*κ*B. Additionally, training-induced changes in nitric oxide levels may also be a mechanism that controls the inflammation induced by 6-OHDA.
